# Targeting PDZ domains as potential treatment for viral infections, neurodegeneration and cancer

**DOI:** 10.1186/s13062-021-00303-9

**Published:** 2021-10-12

**Authors:** Caterina Nardella, Lorenzo Visconti, Francesca Malagrinò, Livia Pagano, Marianna Bufano, Marianna Nalli, Antonio Coluccia, Giuseppe La Regina, Romano Silvestri, Stefano Gianni, Angelo Toto

**Affiliations:** 1 Istituto Pasteur - Fondazione Cenci Bolognetti, Dipartimento di Scienze Biochimiche “A. Rossi Fanelli” and Istituto di Biologia e Patologia Molecolari del CNR, Sapienza Università di Roma, 00185 Rome, Italy; 2 Laboratory Affiliated with the Institute Pasteur Italy - Cenci Bolognetti Foundation, Department of Drug Chemistry and Technologies, Sapienza University of Rome, Piazzale Aldo Moro 5, 00185 Rome, Italy

**Keywords:** Inhibitors, Small molecules, Protein–protein interactions

## Abstract

The interaction between proteins is a fundamental event for cellular life that is generally mediated by specialized protein domains or modules. PDZ domains are the largest class of protein–protein interaction modules, involved in several cellular pathways such as signal transduction, cell–cell junctions, cell polarity and adhesion, and protein trafficking. Because of that, dysregulation of PDZ domain function often causes the onset of pathologies, thus making this family of domains an interesting pharmaceutical target. In this review article we provide an overview of the structural and functional features of PDZ domains and their involvement in the cellular and molecular pathways at the basis of different human pathologies. We also discuss some of the strategies that have been developed with the final goal to hijack or inhibit the interaction of PDZ domains with their ligands. Because of the generally low binding selectivity of PDZ domain and the scarce efficiency of small molecules in inhibiting PDZ binding, this task resulted particularly difficult to pursue and still demands increasing experimental efforts in order to become completely feasible and successful in vivo.

## Introduction

Proteins take part in almost all biological processes, and exert their functions in different ways, ranging from enzyme catalysis to mediating the recognition of other molecules, such as small ligands, or binding nucleic acids or other proteins. Protein–protein interactions (PPIs) represent a key event for several physiological cellular pathways of living organisms including gene expression, cell growth, proliferation, nutrient uptake, metabolism, morphology, motility, intercellular communication and apoptosis.

PPIs are mediated by structural domains with the function to recognize and bind specific sequences on other proteins. These interaction domains are grouped into families and classes, based on structural features, sequence homology and the ability to recognize and interact with specific motifs and sequences [1, 2]. For example, phosphotyrosine containing motifs are mainly recognized by SH2 and PTB domains [3–5], while phosphoserine/threonine containing sequences by 14-3-3 proteins, FHA domains, WW domains and WD40 domains [6]. Acetylated or methylated lysine residues are specifically recognized by Bromo and Chromo domains, respectively [7–9]. Other domains that do not bind post-translationally modified sequences, recognize peptide motifs carrying other features. For instance, SH3, WW, EWH1 domains recognize proline-rich sequences [10–12]. Furthermore, other interaction domains so far known mediate specific cellular physiological functions, such as apoptosis (DD, DED, CARD, BH 1-4 domains) [13–16], vesicle trafficking (GYF, Snare, VHS domains) [17–19] and dimerization (SAM domain) [20].

Among PPIs modules, PDZ domains are the largest class in the human proteome, with around 274 PDZ domains identified in 155 proteins [21]. PDZ domains are often present in multidomain scaffold and anchoring proteins, involved in the formation of transient complexes that support several cellular processes such as protein trafficking, signal transduction, cell–cell junctions, and cell polarity and adhesion. Hence, due to their relevant physiological role, whether interactions mediated by PDZ domains are dysregulated, a pathological state often occurs in the cell.

Given these premises, the modulation and inhibition of PDZ domains-mediated protein interactions are attractive targets in the field of drug discovery. Overall, the knowledge about different PPIs and domains involved in protein interaction is essential not only to understand molecular mechanisms at the basis of the cellular processes, but it is also important to clarify the development of disease states in which PPIs are implicated. Noteworthy, different strategies are applied to develop inhibitors directed against certain PPIs whose function can be altered in certain pathological conditions. In this review work, we recapitulate the implication of PDZ domains in diseases such as cancer, cystic fibrosis, neurological disorders and viral infections. In particular, the attention will be focused on those PDZ domains identified as potential therapeutic targets and on the strategies conceived to hijack their dysregulated interactions.

### PDZ domains structure and function

**Fig. 1:**
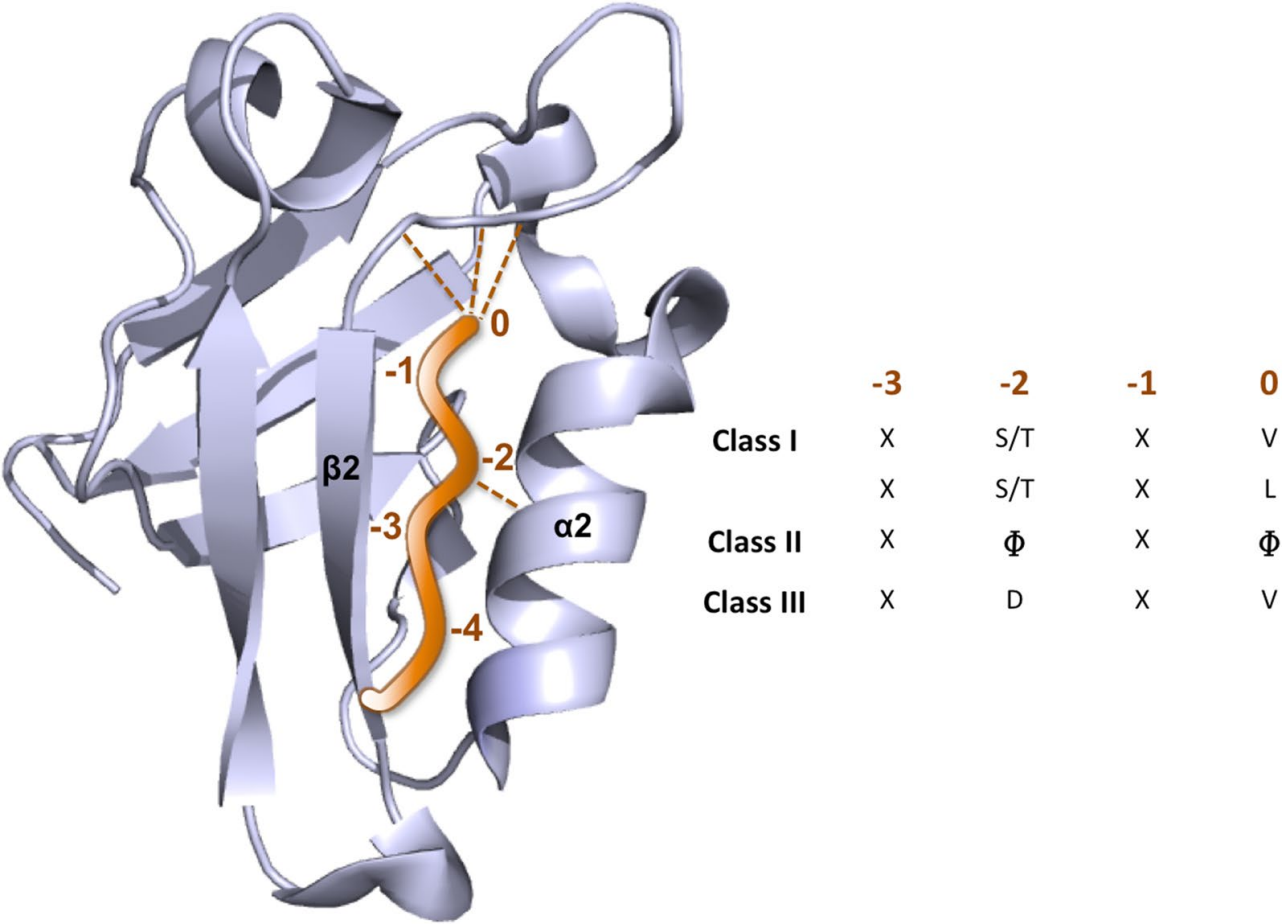
Structure of a PDZ domain (in grey) in complex with a generic C-terminus of a ligand (in orange). The ligand is embedded between the α2 and the β2 of the PDZ binding groove. PDZ Binding Motif positions are highlighted as 0, -1, -2, -3 and -4. The conventional three binding classes are shown in the table in which X corresponds to an unspecified amino acid and Φ to a hydrophobic residue

PDZ domains are small protein modules containing approximately 80–110 amino acids folded in a compact tertiary arrangement, typically consisting of six antiparallel β-strands and two α-helices, with few exceptions. They take their name from Postsynaptic density-95, Disks-large and Zonula occludens-1 proteins, in which they were identified for the first time in the early 1990s [22–24]. Usually, PDZ domains bind the C-terminal sequence of their cognate partners. Ligand binding occurs in a groove formed by the α2 helix and the β2 strand, and the chain of the C-terminal residue of the ligand engages into a hydrophobic pocket on the surface of the domain [25, 26] (as shown in Fig. 1). The binding groove is characterized by the presence of a highly conserved lysine or arginine residue that interacts with the carboxylate group at the end of the peptide. In addition to this electrostatic interaction, three main-chain amide protons of the Gly-Leu-Gly-Phe motif form hydrogen bonds with the C-terminal region of the peptide leading to the more general binding signature R/K-XXX-GLGF.

Conventionally, the C-terminal amino acid of the ligand is numbered as position 0 and the rest of the residues are named in reverse order as position -1, -2, -3 etc. Based on the PDZ binding motif (PBM) of the ligand, three major binding classes have been identified, although lately it has been further expanded into a total of 16. Even if PDZ domains recognize more than 5 residues upstream of the C-terminal residue, the P0 and P-2 positions are particularly significant for specificity. Position 0 is frequently a hydrophobic residue, according to differences in shape and size of the PDZ binding pocket, as in the case of PDZ1 and the PDZ2 of PSD-95, able to recognize sequences ending with valine, leucine and isoleucine [25, 27]. Position -2 is crucial for binding specificity and determines the basis of the entire classification. Class I is characterized by the presence of a serine or a threonine at position -2, with the formation of a hydrogen bond with the N3 of an histidine residue which is highly conserved in the PDZ α2 helix. Class II presents hydrophobic residues at the position -2. Class III recognizes a tyrosine in the PDZ binding groove, establishing a hydrogen bond with the aspartate carboxylic group of the ligand. PBMs can also occur in internal sequences, and membrane phospholipids can be recognized by PDZ domains [28, 29]. As for example, neuronal nitric oxide synthase (nNOS) and the PDZ domain of syntrophin [30] interact through a nonterminal hairpin turn of nNOS in an unusual head-to-tail arrangement.

## Role of PDZ-containing proteins in human diseases

PPIs mediated by PDZ domains have a critical role in the regulation of a broad variety of biological processes. PDZ domains are crucial in the assembly of the molecular machinery of different transduction pathways being often found in multidomain scaffold proteins implicated in the regulation of pre- and post-synaptic signalling in neuronal cells, maintenance of cell–cell junction communication and ion-channel trafficking regulation. Dysregulation of such processes is at the basis of several disorders and diseases highlighting PDZ domains as promising drug targets.

### Cancer

PDZ domains are present in proteins involved in formation of cellular polarity and signalling complexes as well as in the binding to receptors and in localization of channels and enzymes. Thereby they are mainly located at level of the cellular membranes and cytoskeleton and play a central role in processes requiring cell–cell or protein–protein contacts [31], thus orchestrating para- and intra-cellular pathways. As a consequence, dysfunctional PDZ-containing proteins influence development and progression of cancer diseases by determining loss of cell polarity, cell–cell contacts signaling pathways and controlling proliferation, differentiation and apoptosis [32, 33]. Several tumours such as breast, cervical, colon, prostate, liver cancers and glioblastoma often see the involvement of PDZ domains [34].

#### PDZ domains involved in cell polarity

Cell polarity is a key feature of specialized cells, finely regulated and tuned, both spatially and temporarily, by several PDZ-containing proteins. In general, it relies on the asymmetric distribution of macromolecules, like proteins, lipids and RNA of the plasma membranes of epithelial and endothelial cells and neurons, pinpointing defined regions (namely basal, apical, basolateral) with specific structural and functional features [33].

Cell polarity and communications between adjacent cells are ensured by specific structures named adherent junctions (AJs) and tight junctions (TJs). AJs are prevalently found at the basolateral region and regulate cell–cell adhesion through transmembrane proteins named nectins and cadherins. Tight junctions (TJs) determine the membrane polarity by the action of membrane proteins named occludin and claudin [35], localizing components across the different sides of the membranes and regulating ions and solutes transport among cells. TJs and AJs play a key role in the maintenance of tissue integrity and regulate essential cellular functions, such as proliferation, apoptosis, metabolism, differentiation, and motility.

The link between cell polarity and tumor proliferation processes has been identified for the first time in Drosophila [36]. Most polarity complexes forming TJs and AJs are composed by several PDZ-containing proteins: (i) PAR (partitioning-defective) complex generating TJs are formed by interaction between Par3 and Par6 with the atypical protein kinase C (aPKC) and cell division control protein (CDC42); (ii) CRUMBS polarity complex is composed by the transmembrane protein CRUMBS and the scaffolding PDZ-containing proteins PALS1 e PATJ; (iii) SCRIBBLE complex generating AJs are composed of e-cadherin and Scrib, interacting with DISCS large proteins (Dlg) and Lethal-2-giant larvae proteins (Lgl) [33]. Furthermore, the AJs and TJs are physically linked by the zonula occludens proteins (ZO), and include other PDZ domain-containing proteins as signaling molecules and actin cytoskeletal modifiers [37]. Several of these PDZ proteins belong to the MAGUK family, scaffolding proteins recruiting cellular receptors and signaling molecules.

Since AJ and TJ are distinctive of specialized differentiated cells, disturbances of these complexes were found to be strongly correlated to cancer [33]. In particular, loss of tissue integrity and subsequent increased tendency to invasion and metastasis of cancer cells [38] have been ascribed to dysfunction of Scrib [39], Magi [40, 41], Dlg-5 [42, 43], Patj, Lin7, Par3 and Par6 [44], ZO1 [45], ZO2 [34, 46, 47], and Dlg1 [48]. The latter, being part of the Scribble polarity complex, leads to uncontrolled epithelial cell proliferation and neoplastic transformation.

#### PDZ domains mediate signalling complexes formation

The role played by PDZ domains in recruiting proteins involved in molecular signal transduction complexes implied in cell survival, apoptosis, proliferation and differentiation makes them important pharmacological targets in cancer. The oncosuppressor Pten is able to bind the PDZ domains of the Par3, NHERF and Magi-1 proteins. In particular, the binding of Magi-1 to Pten protects it from degradation [40, 49, 50], down-regulates of the PI3K/Akt pathway [51] and exerts an oncosuppressor activity. Interestingly, in acute lymphoblastic leukemia [52] and in colon cancer the expression of Magi-1 is commonly found downregulated.

**Fig. 2:**
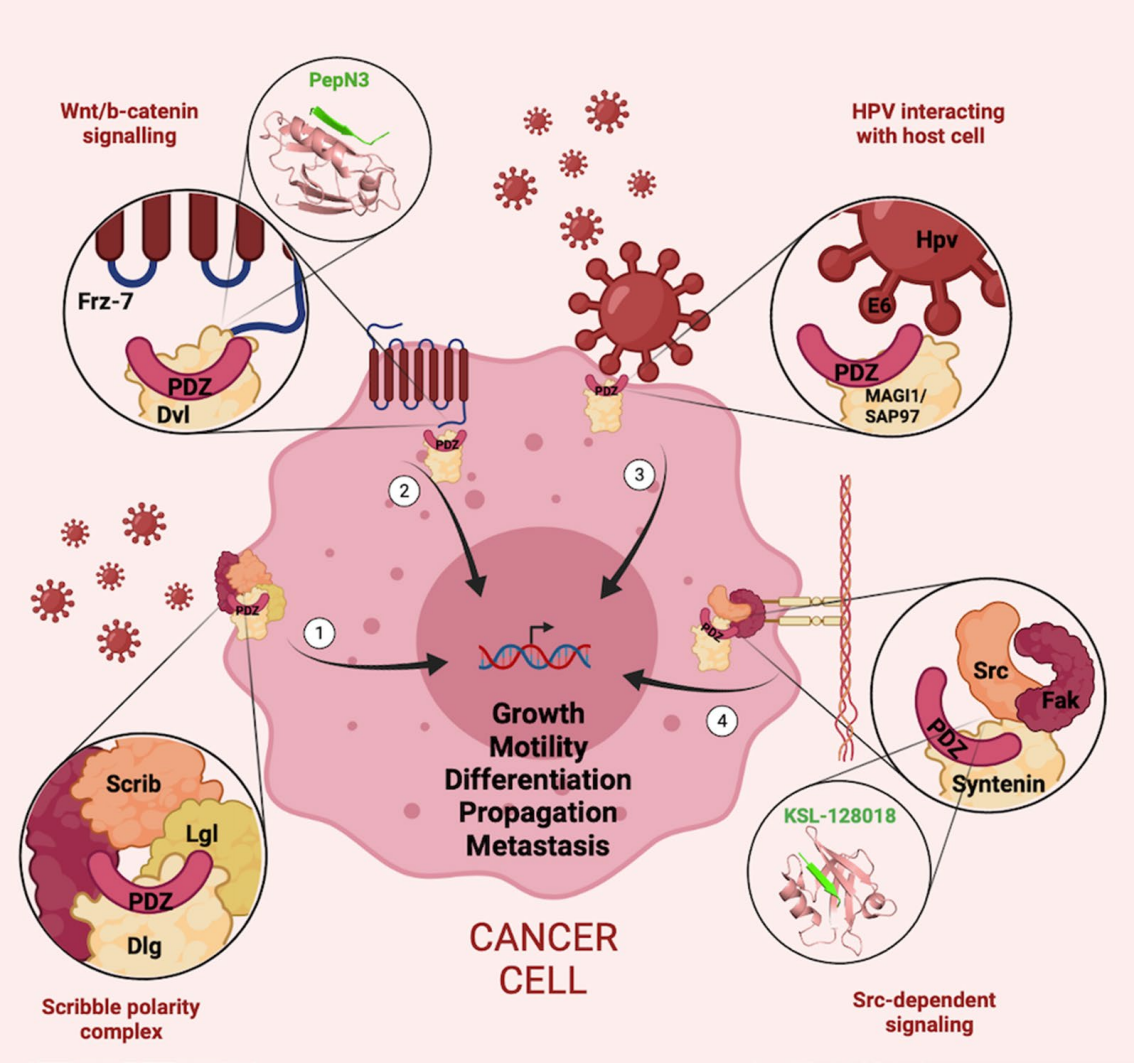
General representation of the cellular localization of the PDZ domains controlling signaling pathways involved in cancer development and progression. (1) Tight junction and Scribble polarity complex. In epithelial cells this complex negatively regulates cell proliferation by inhibiting the expression of the key cell cycle regulator, Cyclin E, and it promotes apoptosis by blocking expression of the apoptosis inhibitor DIAP1. (2) In detail: the Frizzled receptor binds the PDZ domain of Dvl promoting the activation of Wnt signalling, with consequent activation of β-catenin dependent cell proliferation regulating cytoskeletal remodeling and cell migration. As detailed in the text, different inhibitors of Frz-7 and PDZ domain of Dvl interaction were identified and tested. (3) The principal event in tumorigenic activity of E6 protein from High-Risk Human Papillomaviruses derives from its ability to inactivate tumor suppressor p53 protein. The carcinogenic activity of high-risk HPVs is achieved through the interaction between E6 and PDZ domain of SAP-97 and Scribble proteins (4) Syntenin is a PDZ containing protein comprising a tandem of two PDZ domains, involved in cell migration, cytoskeletal rearrangement and metastasis formation. It activates the nuclear factor-kappa B (NF-kB) pathway regulating the expression of genes involved in cell motility and invasion. Monomeric peptide named KSL-128018 is reported as example of inhibitor of PDZ domains of syntenin (see details in the text). (Figure designed through BioRender.com online tool)

Another important example is represented by the PDZ-containing protein Syntenin, also named melanoma differentiation-associated gene-9 (MDA-9). Syntenin expression is increased in metastatic and invasive cells [53, 54]. Numerous studies have shown that syntenin is involved in the formation of metastasis, cell migration and cytoskeletal rearrangement through the activation of the nuclear factor-kappa B (NF-kB) pathway (Fig. 2) [55, 56] which regulates the expression of genes involved in cell motility and invasion [57]. From a structural perspective, Syntenin comprises a tandem of two PDZ domains mediating its activity. Liu and coauthors designed a dimeric ligand binding both PDZ1 and PDZ2 domains with high affinity [58], preventing the interaction with cellular targets. The dimeric inhibitor is based on two sequences naturally interacting with Sytentin PDZ domains, connected each other through a PEG3 (triethylene glycol) linker and modified at positions -2 and -1 with the amino acid naphthyl-alanine. In vitro experiments conducted by Liu and co-workers reported high affinity (K_D_ = 0.21 ± 0.01 µM), together with the ability to reduce cellular migration by downregulating ERK/MAPK pathway *in cellula* (58).

A recent work by Haugaard-Kedström and co-workers reported the design of new monomeric peptides (KLS-128018, KSL-128114) able to bind PDZ1 of Syntenin in a non-canonical way with high affinity. These Peptides showed a promising effect against highly aggressive cancer forms, such as glioblastoma (GBM) [59].

#### PDZ domains bind G protein coupled receptors

G protein coupled receptors (GPCR) are a family of receptors that regulate numerous physiological functions. Several GPCRs are reported as binders of PDZ domains through their PDZ binding motifs (PBMs) [60]. Since the main pathways triggered by GPCRs, PKA and PLC signaling pathways, regulate cell survival, growth, migration and differentiation [61, 62], alterations of the binding between PDZs and GPCRs are often tumorigenic.

A typical example is represented by the Frizzled receptor (Frz). It is involved in the activation of the Wnt signal pathway through the interaction of its C-terminal PBM with the PDZ-containing protein Disheveled (Dvl) [63]. Dvl triggers the activation of two signaling pathways: the canonical pathway activating the β-catenin dependent cell proliferation [64] and the non-canonical pathway regulating cytoskeletal remodeling and cell migration [65]. Both pathways are involved in tumorigenesis [66, 67]. Given the importance of this signalling pathway in the onset of cancer, targeting the PDZ domain of Dvl protein represents a promising antitumor strategy, especially by inhibiting the interaction with Frizzled proteins.

In particular, Frizzled 7 (Frz7) is a receptor subtype highly expressed in a broad range of tumours, exerting its oncogenic activity through Wnt pathway activation. The disruption of this interaction can be selectively addressed by designing inhibitors with peculiar characteristics. Fujii and coworkers [68] proposed a small molecule, called FJ9, which was reported to inhibit the binding between Frz7 and the PDZ domain of Dvl. This inhibition lowered cytosolic level of β-catenin in a dose-dependent manner, downregulating the canonical Wnt pathway in cells and inducing apoptosis in human lung cancer and melanoma cells.

In another work from Mahindroo et al. [69] a small molecule was successfully designed to disrupt the interaction between Frz7 and PDZ of Dvl. The molecule, an indole-2-amide compound, was able to bind the PDZ domain by mimicking the side chains of the second and fourth amino acid residues of the endogenous ligand through the substituents at positions 2 and 3 of the indole. The compound was found to interact with Frz7 and exert an apoptotic effect by down-regulating Dvl-driven Tcf activation of transcription.

Zhang and coauthors have elegantly designed, through phage-display, short peptides mimicking internal ligands binding the isoform 2 of Dvl (Dvl2) able to inhibit the Wnt/β-catenin pathway. Among these peptides (called pepN1, pepN2 and pepN3), pepN3 inhibited the Wnt/b-catenin pathway in a dose-dependent manner with potency (IC_50_ of 11 ± 4 µM) superior to FJ9 (see above in the paragraph) and was atoxic for the cells at active concentrations [70].

Several other proteins, such as LARG-Rho [34], CAR11 [71], GIPC1 [72], NHERF1-2 [73], PREX1 [74], TIAM1 [34], show interaction with GPCRs and are associated to cancer, an aspect reinforcing the view that targeting PDZ mediated protein–protein interactions may be a powerful strategy aimed to develop effective antitumor pharmacological strategies.

### Cystic fibrosis

Cystic fibrosis is a genetic disorder caused by mutations in the *CFTR* gene encoding Cystic Fibrosis Transmembrane Conductance Regulator (CFTR). CFTR protein is a chloride channel activated by local cAMP that is located in the apical membrane of epithelial cells. The activity of CFTR contributes to maintaining the correct state of hydration on the epithelial surface by regulating the flux of ions and fluid through the plasma membrane [75–77]. CFTR is expressed in different organs including lung, intestine, pancreas and kidney. Given its important physiological role, dysregulation of CFTR signaling causes severe defects in these organs. The impairment in the transport of ions through the epithelial membrane leads to an accumulation of thick mucus in the lung that obstructs the airways [78–80].

**Fig. 3:**
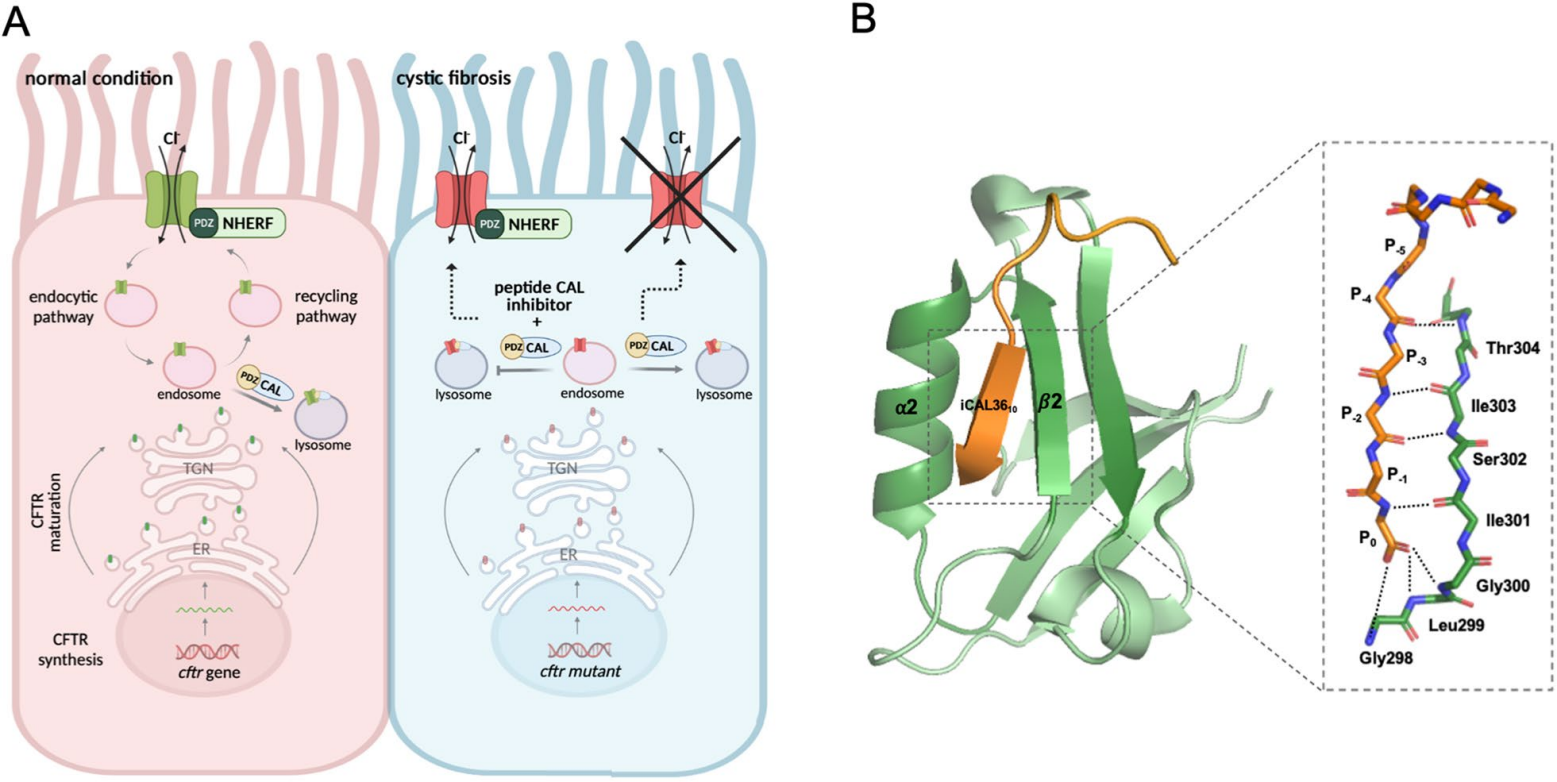
**A** Airway epithelial cells in normal conditions (red) and cystic fibrosis (light blue). Interaction of NHERF and CAL with CFTR mediated by the PDZ domains is shown. In normal condition, NHERF stabilizes CFTR to the plasma membrane, while CAL promotes the CFTR degradation in lysosomes. In cistic fibrosis the ΔF508A mutation in CFTR causes its rapid degradation mediated by CAL, with consequent absence of the CFTR at the plasma membrane. Inhibitors of the interaction of CAL with CFTR could restore the presence of CFTR at the plasma membrane. **B** 3D structure of the complex formed by iCAL36 10 (in orange) and PDZ CAL (in green). The box shows the hydrogen bonds between the main chain functional groups of the peptide with the β2 strand of PDZ CAL. (Figure designed through BioRender.com online tool)

Several PDZ proteins are involved in the regulation of CFTR. In particular, CAL (CFTR associated ligand) has been identified as an important regulator of CFTR endocytic trafficking and lysosomal degradation [81]. CAL is a protein of 461 residues that is ubiquitously expressed. Its structure consists of unstructured (unfolded) N- and C-terminal regions, a coiled-coil domain and a PDZ domain (Fig. 3). The PDZ domain of CAL contains a His residue at the N-terminal of α2 helix which allows it to bind preferentially PBMs of class I. The recognition of a PBM in CFTR and the PDZ domain of CAL promotes the degradation of CFTR in lysosomes, with consequent reduction of the levels of CFTR on the plasma membrane surface [82–84]. Other two PDZ-domain proteins, the two isoforms of Na^+^/H^+^ exchanger regulatory factor (NHERF 1 and 2), have been identified as positive regulators of CFTR. NHERF 1 and NHERF 2 recognize the cytoplasmic tail of CFTR through their two PDZ domains, stabilizing its localization at the plasma membrane and thus regulating its activity [85].

The most common mutation occurring on CFTR gene and causing CF is the deletion of the Phe508 residue (∆F508) [79]. This mutation causes an inefficient folding of the protein and a decreased channel activity, as well as a rapid degradation mediated by the interaction between CFTR PBM and the PDZ domain of CAL [82, 86, 87]. Examples of molecules identified as inhibitors of PDZ-domain mediated interactions involved in the cystic fibrosis will be discussed.

#### Inhibitors of PDZ-domains mediated interactions for the treatment of cystic fibrosis

Small molecules have been designed to induce correct folding and channel activity of ∆F508 CFTR [88, 89]. However, due to the rapid degradation in the cell of the protein mediated by CAL, this approach could be applied only in in vitro experiments. In a different work [90], by knocking down CAL in a bronchial epithelial cell line derived from a CF patient ∆F508, CFTR expression and channel activity has been restored. This result suggested that the inhibition of the interaction between the PDZ domain of CAL and CFTR could represent a potential treatment for cystic fibrosis.

Since both CAL and NHERF proteins recognize CFTR, a specific inhibitor of CAL PDZ – CFTR interaction was designed [91]. Through a combination of library screening and different steps of optimization, a decameric peptidyl inhibitor called iCAL3610 was produced (Fig. 3). This molecule showed high affinity for CAL PDZ (K_i_ = 17.3 ± 4.3 µM) and low affinity for the PDZ-domains of NHERF proteins (K_i_ > 5000 µM for the PDZ1 and PDZ2 of NHERF1; K_i_ > 5000 µM and K_i_ > 3000 µM for PDZ1 and PDZ2 of NHERF2, respectively). Experiments conducted in airway epithelial cell lines from CF patients demonstrated that iCAL3610 was able to increase the half-time of CFTR at the plasma membrane and to enhance the activity of the channel [92].

The use of peptides targeting the PDZ domain of CAL to inhibit its interaction with CFTR resulted as an effective strategy, with some limitations due to low cell-permeability and metabolic stability. In a recent study [93] a peptide called PGD97 was designed to overcome these difficulties. It presents two distinctive regions, a cell-penetrating peptide (CPP) and a binding sequence for CAL PDZ, which forms as a disulfide-cyclized macrocycle and becomes linear in the intracellular environment. *In cellula* experiments demonstrated that PGD97 is able to stabilize ∆F508 CFTR and improve CFTR functions [93], representing a potential novel treatment for cystic fibrosis.

### Nervous system disorders

Neuronal network is finely regulated during synaptic transmission. In the synapses, neurotransmitter transporters and receptor complexes are localized in the presynaptic and postsynaptic region respectively, and a fine regulated control is necessary to guarantee the correct propagation of the signal [94]. Abnormal synaptic transmissions are at the origin of a plethora of different pathological conditions. In this scenario, receptor complexes represent the site of action of almost 40% approved drugs, highlighting the importance of these proteins as pharmacological targets [95, 96].

Among the others, PDZ-containing proteins are particularly important for the formation, function and localization of postsynaptic receptor complexes [97, 98]. PDZ proteins can interact not only with membrane proteins but also with cytoplasmic proteins producing a large variety of signalling complexes [99, 100]. Remarkably, PDZ-containing proteins are also involved in the regulation of synaptic adhesion and maturation of excitatory synapses, as exemplified by the case of PSD-95 [97, 101, 102]. Their abundance and crucial role in the regulation of different neurological processes, establish a close link between PDZ dysfunction and the onset of different pathological conditions. In this paragraph we will focus our attention on the role of PDZ domains in the onset of different neurological pathologies [103].

#### Autism spectrum disorder (ASD)

ASD is a neurodevelopmental condition that affects childhood population on average 1 in 68 [104]. Autistic patients are characterized by behavioural abnormalities such as reduced vocal communication, aberrant social interactions and repetition [104, 105], caused mainly by impairments in synaptic plasticity and synaptic processing.

**Fig. 4:**
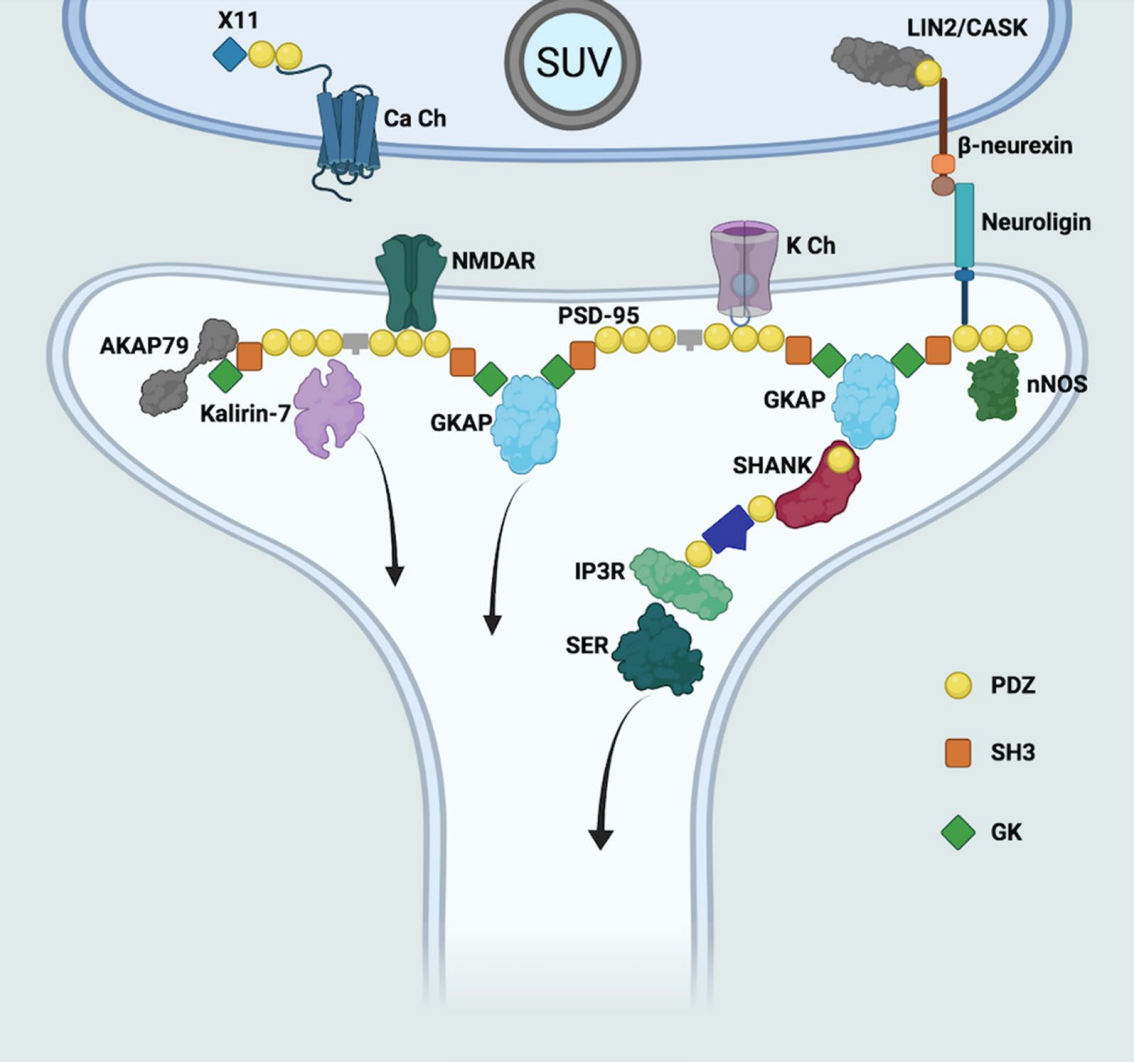
The main PDZ-containing proteins of a glutamatergic synapse are highlighted. PDZ domains are indicated by yellow circles while SH3 and GK domains are represented as orange squares and green rhombuses, respectively. All the other proteins are shown as simple shapes and are labelled. A subset of known protein–protein interactions is illustrated. Ca Ch, calcium channel; nNOS, neuronal nitric oxide synthase; GKAP, guanylate kinase-associated protein; K Ch, potassium channel; SHANK, SH3 and ankyrin repeat-containing protein; IP3R, IP3 receptor; SER, smooth endoplasmic reticulum; PSD-95, postsynaptic density protein 95; NMDAR, NMDA (N-methyl-D-aspartate) receptor; AKAP79, A-kinase anchor protein. (Figure designed through BioRender.com online tool)

Synaptic plasticity is mainly controlled at the level of the postsynaptic density (PSD) region [106]. PSD-95 is one of the major components of PSD and it is composed of three N-terminal PDZ domains and an SH3-GK module [107–109]. It is able to interact, through its PDZ domains, with NMDA receptor subunits, NR2A and NR2B, and with AMPA receptor accessory proteins [107], and it plays a key role in regulating plasticity of glutamatergic synapses (schematized in Fig. 4) during neurodevelopment [105]. As a prevalent component of the postsynaptic density region, the disruption of PSD-95 physiological interactions has been correlated with the onset of ASD [105, 110–112].

Additional evidence of aberrant interactions between PSD-95 and PDZ-containing SHANK family proteins corroborates the link between PSD-95 and ASD [105, 113]. SHANK proteins are scaffolding proteins involved in the formation of large complexes crucial for synaptic development and function. An increasing amount of data indicate that mutations in the genes that encode for SHANK proteins may be correlated with several behavioural abnormalities in mice models, some of which are considered ASD-like behaviours [113–118]. This evidence points the attention on PDZ-containing proteins as targets to develop pharmacological strategies against ASD.

#### Alzheimer’s disease (AD)

AD is a neurodegenerative condition that is becoming dramatically relevant in terms of health care and welfare in the modern world [119, 120]. A peculiar characteristic of AD is the accumulation of protein aggregates (or plaques) in the brain tissue [120–122]. Principal components of AD plaques are the Amyloid beta (Aβ) peptides, natural products of amyloid precursor protein (APP) metabolism [121]. APP is a transmembrane protein of 695–770 amino acids, cleaved by the action of β- and γ-secretases that generate Aβ fragments of various lengths. The Aβ_1-42_ variant displays a high propensity to form insoluble aggregates.

One of the major interactors of APP is Munc18-interacting proteins (Mints or X11s) [123–125]. Mint proteins present a variable N-terminal region, and a conserved C-terminal region that contains a phosphotyrosine binding (PTB) domain and two PDZ domains. Interestingly, PDZ domains of Mint are regulated by an intramolecular mechanism, in which the highly conserved C-terminal tail of Mint is able to inhibit the binding of the construct [126]. The PTB domain binds the conserved YENPTY motif of APP controlling its trafficking and regulating Aβ fragments production [127, 128]. Additional evidence about the involvement of Mint in the onset of AD derives from the description of the interaction between its PDZ domains and Presenilin-1 [129–131], the catalytic subunit of the γ-secretase complex. The interaction between the PDZ domains of Mint with Presenilin-1 promotes APP/Presenilin-1 colocalization regulating Aβ fragments formation. Altogether, these findings indicate that APP/Mint/Presilin-1 interaction is of great interest for potential treatment of AD, with PDZ modulation as a feasible therapeutic target.

#### Parkinson's disease (PD)

PD is a neurodegenerative disorder affecting 2–3% of the population over 65 years old. PD is the most common neurodegenerative movement disease, characterized by a loss of dopaminergic neurons in the substantia nigra and the accumulation of protein aggregates called Lewy bodies [132, 133]. The main constituent of Lewy bodies is α-synuclein protein, which tends to form aggregate as a consequence of misfolding [120, 134].

The molecular mechanism of α-synuclein misfolding has been extensively investigated, but still poorly understood. One of the prevalent causes of protein misfolding is the exposure of cells to internal and external stress which cells tend to contrast through different strategies. An example is represented by the oligomeric HtrA protease family [135]. HtrA protease contains one or two C-terminal PDZ domains, which bind misfolded proteins, triggering the activation of the trypsin-like protease domain for degradation [135–137]. A direct correlation between the mammalian HtrA2 inactivation and the onset of PD has been established in different studies [138–140], and PD patients have been identified through missense mutations in the gene encoding for HtrA [138, 139]. Interestingly, one of these mutations, p.G399S, affects one of the PDZ domains of HtrA2 [139]. Other studies performed on HtrA2 deficient mice exhibiting neurodegeneration and a parkisonian phenotype [140, 141] report additional evidence of the correlation between HtrA2 inactivation and the onset of PD.

#### Hearing and vision diseases

Hearing and vision mechanisms control is mainly achieved by clustering signaling proteins in large molecular complexes, the organization of which foresees the action of many PDZ-containing proteins.

Usher syndrome (USH) is a syndrome affecting hearing, vision and body balance, and represents the most common form of hearing and vision loss [142]. PDZ domains play a pivotal role in the onset of this pathology. Two pioneering works by Verpy and colleagues and Bitner-Glindzicz and colleagues described mutations in a gene encoding the PDZ-containing protein Harmonin [143] that lead to Usher type 1C (USH1C) disease [144].

Usher syndrome can occur in three distinct forms with USH type 2 (USH2) as the most common. Among the others there are three genes that are recognized to cause USH2 named USH2A, GPR98, and WHRN, whereas a fourth gene, PDZD7, is a modifier gene found in USH2 patients [145, 146]. Two of these genes, WHRN and PDZD7, codify for PDZ-containing proteins able to mediate the formation of a quaternary complex with the other two proteins codified by USH2A and GPR98 [145]. Inactivation and mutations in the PDZ domains of Whirlin and PDZD7 proteins have been associated directly to the manifestation of USH2 [143, 145, 147].

#### Ischemic stroke

Ischemic stroke represents the leading cause of disability and the second cause of death worldwide, with an urgent need for pharmacological therapies able to relieve neuronal damages [148]. The interaction between the PDZ domains of PSD-95 and the N-methyl-D-aspartate (NMDA) receptor is a key step in the onset of stroke. In particular, this interaction causes an overproduction of nitric oxide and consequent neuronal death [149]. On these bases, a suitable candidate for the treatment of neuronal damage after stroke is represented by hijacking the interaction between the PDZ domains of PSD-95 and NMDA.

#### Targeting PDZ domains as potential neurological treatment

PDZ domains represent an extremely interesting candidate for novel neurological treatments. A peculiar feature of PDZ domains is their presence as tandem repeats [150–152]. Interfering with multiple PDZ domains simultaneously can enhance both affinity and selectivity of a putative inhibitor and the design of multivalent ligands to target protein–protein interactions is a promising strategy for the treatment of neurological disorders.

An important example is NA-1 (now in phase 3 of the clinical trial NCT04462536) which targets PDZ domains of MAGUK family proteins (PSD-95, PSD-93, SAP-97 and SAP-102) [153]. The MAGUK structural architecture is generally composed by a tandem repeat of PDZ domains followed by a SH3 and a GK domain. MAGUK proteins are responsible for the formation of post-synaptic protein complexes and are potential drug targets for the treatment of different neurological pathologies. Since the interaction between PSD-95 and N-methyl-D-aspartate receptor (NMDA) and neuronal NO synthase (nNOS) is mediated by PSD-95 PDZ domains, Nissen et al. designed and produced a trimeric ligand able to bind simultaneously PDZ1-2–3 domains of PSD-95. NA-1 showed high binding affinity towards these MAGUK proteins, confirming the therapeutic importance of this PDZ-protein interaction.

Another example of a potential treatment of dysfunctional neurological conditions obtained by inhibiting PDZ domain-mediated interaction is the Tat-P4-(C5)2 peptide, studied in vivo by Christensen and coworkers [154]. To avoid direct targeting and inhibition of NMDA and AMPA receptors, which could lead to significant side effects, a peptide with the ability to interfere with synaptic PDZ domain-scaffold protein PICK1 was developed. PICK1 regulates the expression and activity of AMPA receptors and mediates important membrane protein interactions, including those with the GluA2 subunit of AMPA. Tat-P4-(C5)2 resulted in being able to block two different regions of the PICK1 PDZ domain, leading to the disruption of the interaction with AMPARs, and consequently, limiting the dysfunctional synaptic plasticity associated with correlated neurological disorders, such as neuropathic pain.

### Viral proteins targeting PDZ domains

A large number of PDZ-containing proteins possess a fundamental role in maintaining the correct physiology of the cell and in determining key aspects of specialized cells, as for example cell polarity in epithelial and neuronal cells. Under this light, it is not surprising that several viral pathogens have evolved PDZ-binding motifs in their proteins to target host cell PDZ domains and disrupt physiological interactions to favor viral replication and disease progression.

At the end of 1990s, the first PBMs in viral proteins were identified in HTLV-1 Tax protein [155], high-risk HPV E6 oncoproteins [156], and adenovirus E4 oncoprotein [157]. Since then, many other viral proteins were found to present PBMs in their sequence and the evolutionary strategy of viral pathogens to target these protein–protein interactions became clearer. In fact, a wide range of pathologies may arise from viruses that developed the ability to target PDZ protein functions and many well-established PDZ targeting viral proteins have been pinpointed and characterized [155, 158–163] reinforcing the evidence on how PDZ domains represent fundamental pharmacological targets in the struggle to prevent and cure several viral diseases.

#### PBM of Coronaviruses Envelope protein

A typical example of viral protein targeting PDZ domains upon infection is represented by coronaviruses MERS-CoV, SARS-CoV, and SARS-CoV-2 Envelope (E) membrane proteins. The genome of coronaviruses typically encodes for four major structural proteins, including spike (S), nucleocapsid (N), membrane (M), and envelope (E) proteins. The E protein is a tiny integral membrane protein that is poorly present in the mature virus, while being highly expressed in the host cell, where it forms, through interaction of their transmembrane domain, pentameric ion channels mainly involved in virion maturation and trafficking [164].

E proteins from SARS-CoV and SARS-CoV-2 possess a PBM at their C-terminus which allows them to interact with PDZ-containing proteins in the host cell. Their primary structures have 98% sequence identity. The few variations appreciable between the two protein sequences occur at their C-terminal domain and do not affect the PBM. Although the exact role of these interactions is not well established, there is evidence about SARS-CoV E protein PBM as virulence factor [165]. *In cellula* experiments reported that the removal of the PBM from SARS-CoV E protein resulted in attenuated virulence. Furthermore, the acquisition of new alternative PBMs after a number of cell passages confirmed the key importance of the binding with PDZ proteins for the virus activity. Interaction occurring between SARS-CoV E protein and PDZ domains of PALS1 and syntenin [166] as well as with other PDZ-containing proteins such as TJP1-2, PTPN13, HTRA1, MLLT4, PARD3, LNX2, recognizing the PBM of SARS-CoV E protein, and NHERF1, MAST2, RADIL, SNX27 as specific interactors of SARS-CoV-2 E protein [167] has been reported.

The interaction of E protein with PALS1 is of particular importance. PALS1 is a member of MAGUK superfamily and it is the human homologue of D. melanogaster Stardust protein. It is composed of two N-terminal L27 domains, followed by a PDZ domain, a SH3 domain and a guanylate kinase domain (GUK). PALS1 possesses a central role in determining the Crumbs polarity complex, which, in its entirety, is composed of a transmembrane protein (Crumbs) and two cytoplasmic scaffolding proteins, PATJ and PALS1. The association of PALS1 with Crumbs is mediated by PDZ, SH3 and GUK domain supramodular structure [168], determining the apical portion of epithelial cells, together with the interaction with PATJ and MUPP1 [169, 170]. The interaction between PALS1 PDZ domain and the E protein of SARS-CoV (and SARS-CoV-2) is suggested to be at the basis of lung epithelial damage [163, 165], retaining the protein in Golgi vesicles after synthesis, and preventing the correct formation of tight junctions. As a result, a diffuse damage of lung epithelial tissue occurs, representing one of the most dramatic and fatal outcomes of coronavirus infections. Given these premises, reinforced by the global health emergency due to SARS-CoV-2 spread and the severity of COVID-19 pandemic, the PDZ domain of PALS1 represents a strategic site to be pharmacologically targeted.

The three dimensional structure of the PALS1:E protein complex has been recently resolved [171] and outlined a synergistic mechanism of ligand recognition exerted by PDZ and SH3 domains. Recent studies showed a higher affinity of E protein from SARS-CoV-2 compared to SARS-CoV for the isolated PDZ domain of PALS1 [172, 173]. Molecular Dynamics simulations highlighted the SARS-CoV-2 Arginine 69 as key residue responsible of the improved affinity to PALS1 compared to the SARS-CoV homologue, in particular by enhancing polar interactions with negatively charged pockets of PALS1 PDZ domain, resulting in significantly reduced mobility of the viral protein. These data supported the hypothesis that the typical virulence of SARS-CoV-2 may rely on the improved binding of E protein with PALS1 and represent a first step in understanding the mechanistic details of this important interaction, which should be further investigated to develop potential SARS-CoV-2 antiviral strategies.

#### Oncogenic viral PDZ domain interacting proteins

As detailed in paragraph *2.1*, a large number of PDZ-containing proteins possess key roles in the maintenance of the correct physiology of the cell at different levels, ranging from transcription factors to scaffolding proteins and to polarity determination of specialized cells. Consequently, viruses expressing proteins able to target PDZ domains are often correlated with the onset of cancer pathologies.

There are several examples of viruses exerting their oncogenic activity by targeting PDZ containing proteins, as for example Hepatitis C virus [158], Hepatitis B virus [159, 160] and Human T Cell Leukemia Virus 1 [155, 161]. However, one of the most studied and characterized PDZ interacting viral proteins is the E6 protein from HPV (Human Papillomavirus) (Fig. 2). Despite over 200 different strains of Papillomaviruses being identified, only a small subset (around 30) of them are reported as carcinogenic, denoted as high-risk HPVs. Interestingly, only high-risk HPVs contain a PDZ-binding motif at the C-terminus of their E6 protein, highlighting the importance of PDZ mediated interaction in the tumorigenic progress of these viruses infection.

The principal event in tumorigenic activity of E6 is its ability to inactivate tumor suppressor p53 protein [174, 175]. Furthermore, many PDZ proteins with key roles in cell proliferation, cell growth, cell polarity and protein degradation are reported as interactors of E6 high-risk HPVs [176]. For such reasons, a number of studies have focused on understanding the binding process of E6 proteins with PDZ domains and the molecular details of this interaction [176]. In general, carcinogenic activity of high-risk HPVs is achieved through the interaction between E6 and PDZ domain of SAP-97 and Scribble proteins. Recently, the ability of high-risk HPV-16 and HPV-18 E6 proteins to target the PDZ domain of NHERF2 has been reported [177]. NHERF2 is a tumor suppressor protein which, among other functions in the cell, regulates endothelial proliferation. Since in transformed cells apoptosis is prevented only as long as the expression of E6 and E7 are sustained in the host cell, PDZ domains interacting with E6 proteins represent promising drug targets to contrast the tumorigenic activity of high-risk HPVs.

#### Protein-Engineering optimized PDZ domain as pharmacological strategy

With the final goal to bind and sequester E6 protein from high-risk HPV18 and prevent it to interact with host cell targets, a recombinant optimized PDZ domain was conceived and produced (named "PDZbody") by designing a chimeric domain composed of a strategically mutated PDZ domain fused with an extra alpha helix from E6AP (E6-associated protein), a different interactor of E6 protein in the host cell [178]. This engineered PDZbody allowed to target two different sites of the E6 proteins, with an increased affinity compared to its natural interactors [178]. This strategy reported promising results both in in vitro and *in cellula* experiments, confirming the ability of PDZbody to trap E6 protein from interacting with host cell proteins. By following an analogous approach, a protein chimera composed of the PDZ2 domain of the tight-junction protein MAGI1 and the LxxLL motif of E6AP was designed to disrupt E6 interactions in the host cells [179]. Although these strategies represent an important starting point in the attempt to hijack the oncogenic activity of high-risk HPVs, the diversity of sequences of E6 proteins among the high-risk HPVs family and the several PDZ domains involved in the pathogenic pathways makes an arduous effort to develop focused pharmacological therapies and further experimental efforts are demanded in order to step forward to this goal.

## PDZ Inhibitors

The crucial role played by PDZ domain in the spreading of cellular signals and their aberrant activations in many human diseases makes the PDZ domains very attractive targets for drug discovery (a summarizing list of PDZ-containing proteins involved in human pathologies is reported in Table 1). Thus, the discovery of small molecules able to impar the protein–protein interactions in which PDZs are involved, proved to be very profitable, rather than targeting entire signaling cascades, as typically achieved by receptor antagonists. Efforts in the identification of modulators of the PDZ signals has widely increased in the last years. Since 1996, when it was reported the first peptide able to bind a PDZ domain [180] more than 600 papers about PDZ inhibitors were published (source SciFinder). Among them, many remarkable results were reported such as the PDZ inhibitors of PSD-95 [181], Synthenin [59] and Dvl [69, 70]. More than 600 crystal structures of human PDZ domains available at the protein data bank (ww.rcsb.org) have brought significant insight into the rational design of PDZ modulators. This structural information encouraged the use of molecular modelling studies. Virtual screening is highly used to identify PDZ inhibitors; indeed, it is possible to use the cognate substrate of the targeted PDZ to design a pharmacophore model by which selecting the studied compounds [182–184].

**Table 1:**
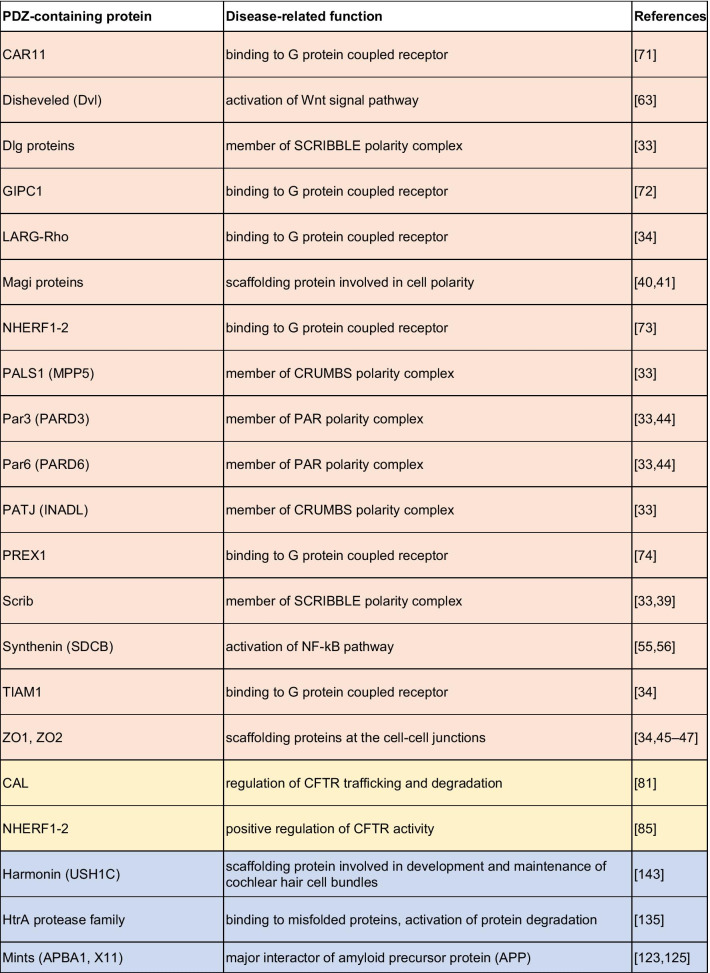

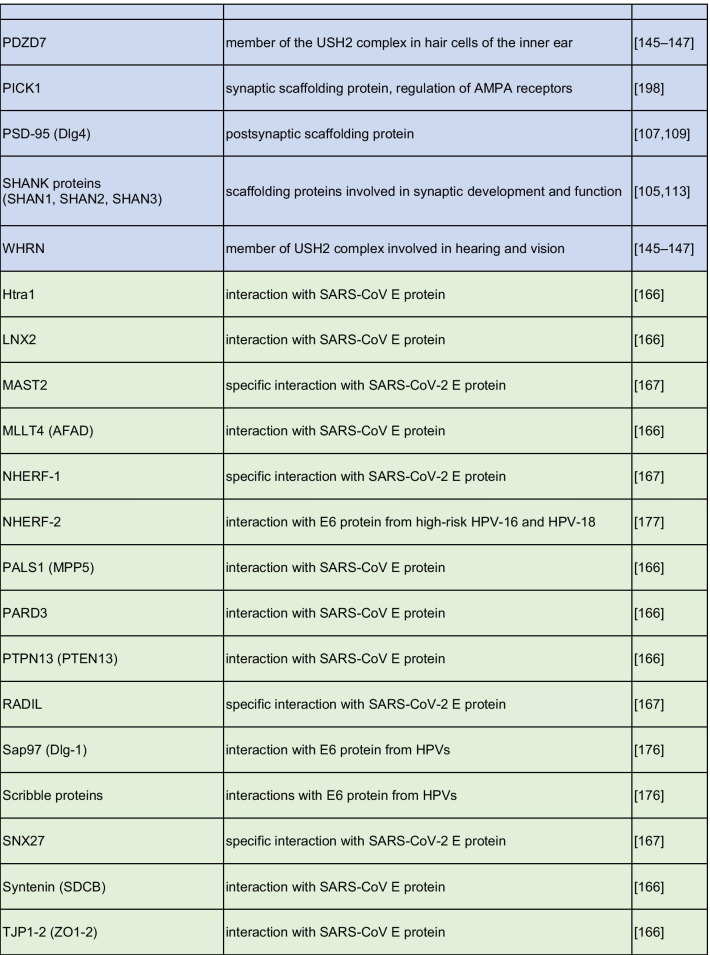
A list of major PDZ-containing proteins involved in human cancer diseases (light red), cystic fibrosis (yellow), nervous system disorders (light violet), viral infections and cancer induced by viruses (light green)

**Fig. 5:**
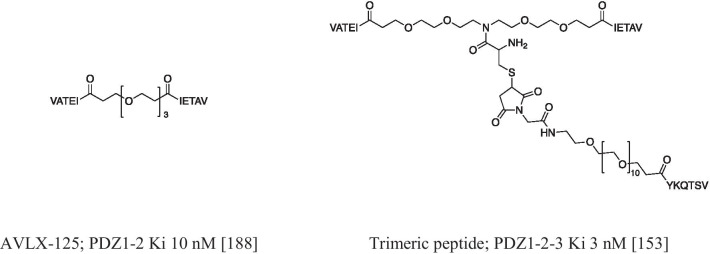
Chemical structures of AVLX-125 inhibitor molecule and its trimeric peptide derivative targeting PSD-95 PDZ domains

Despite the promising results, at the state of the art, no small molecules are in clinical or preclinical phase. It may be due to: (i) the wider surface of the binding pocket, making difficult to reach with high affinity the binding site by a small molecule [71, 185]; (ii) the promiscuity of the PDZ domains making selectivity a big concern [186]; and (iii) the post translational modifications, such as phosphorylation, which have shown to be potent modifiers of binding affinities to PDZ domains and can either decrease or enhance affinity [186]. On the other hand, a large number of peptides and peptidomimetics have been evaluated as PDZ modulators. The most successful story is about NA-1. It is a peptide inhibitor of the PDS-95 PDZ2 with Ki of 4.4 μM. [149]. NA-1 (NH_2_-YGRKKRRQRRRKLSSIESDV-COOH) was designed as a fusion peptide bearing a PDZ binding sequence obtained from the cognate substrate GluN2B NMDAR and a Tat peptide to increase the cell permeability. In phase II clinical studies, NA-1 reduced the number of heart attacks in patients at risk of embolic stroke [187]. Worthy of note, the studies about PSD-95 led to a new paradigm in the design of PDZ inhibitors. The very similar structures of PDZ1 and PDZ2 suggested the development of a tandem binder. Starting from the cognate substrate GluN2B, a pentapeptide (IETAV) was identified which dimerized through a PEG linker leading to derivative AVLX-125, with 145-fold improved binding affinity compared with the monomer. and a Ki of 10 nM [188]. Later, the same authors reported a trimeric peptide by adding another branch to AVLX-125. The added peptide (YKQTSV) was different from the previous one, because the PSD-95 PDZ3 had a lower homology with the other PDZ domains. The tripeptide showed a higher affinity compared to the dimer with a Ki close to 3 nM [153] (Fig. 5).

**Fig. 6:**
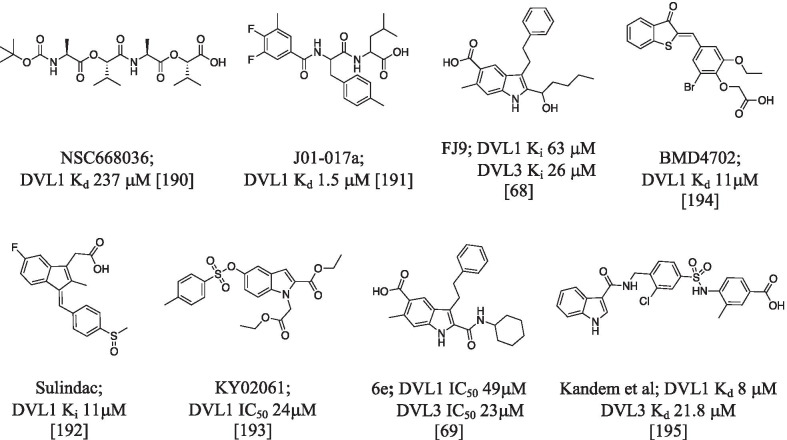
Chemical structures of different DVL proteins inhibitors

The crucial role played by DVL in the cancer development, progression, metastasis and chemotherapy resistance [189] prompted great effort in the development of small molecules able to impair this aberrant PDZ dependent activity. As a result, a large number of DVL small molecule inhibitors were synthesized. The first one, NSC6680036 reported in 2005 [190], was discovered by a structure based virtual screening and the mechanism of action was confirmed by NMR studies [190]. NSC6680036 derivatives were not further developed because of their peptidomimetic rather than small molecule strcuture. Later, the same authors reported derivative J01-017a, identified by virtual screening and 3D QSAR methods, with improved binding affinity (K_d_ 1.5 μM) [191] and FJ9 (DVL3 K_d_ 26 μM) [68]. The latter derivative had an indole/indene-carboxylic acid scaffold which was conserved, with minor modification, in many DVL PDZ inhibitors, such as the non steroidal antinflammatory drug sulindac (K_d_ 11 μM) and KY02061 (IC_50_ 24 μM) [192]. In general, nonsteroidal anti-inflammatory compounds showed the ability to suppress the Wnt pathway in breast, colon and lung cancers [192]. KY02061 identified by virtual screening approach, impaired the Dvl–CXXC5 interaction by binding the PDZ domain, and increased of the β-catenin nuclear concentration favoring the bone anabolic activity [193]. Mahindroo et al. [69] reported an indole based DVL PDZ inhibitor designed by mimicking the peptide sequence of the substrate. The most active compound, 6e, an indole-2-carboxamide-5-carboxylic acid derivative, showed IC_50_ values of 23 and 49 μM for DVL3 and DVL1, respectively (Fig. 6).

**Fig. 7:**
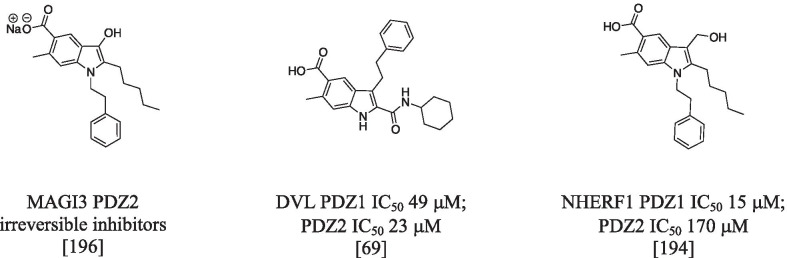
Chemical structures of chemical compounds targeting MAGI3, DVL and NHERF1

Benzofused heterocycles were reported as privileged scaffolds for the inhibition of PDZ of the DVL: indole and indazole with K_d_ in the one-digit micromolar range for DVL1 by Kandem (10.5194/mr-2-355-2021), and benzothiophenone derivatives (i.e. BMD4602 with Kd of 11 μM) by Choi [182]. The indole ring seems to fit well in the binding pocket of the PDZ. Generally speaking, the benzofused scaffold (benzene plus a 5-menber heterocyclic ring) allows to generate a variety of diverse compound libraries with potential high binding affinity and selectivity towards the PDZ family. Indole derivatives were also reported as inhibitors of some other PDZ domains [183]. Representative examples are reported in a series of papers by Fujii et al. By docking analysis the authors identified an irreversible inhibitor of membrane-associated guanylate kinase 3 (MAGI3) PDZ2. The biological activity of this inhibitor assayed in HTC116 cells and the binding was confirmed by mass spectrometry experiments [196]. Introduction of appropriate substituents at position(s) 1, 2 and 3 of the indole ring shifted the targeted PDZ from MAGI3 to DVLs (6e) [69] (for examples, see Figs. 6 and 7). A derivative with different substituent at the indole showed high binding affinity for both PDZ domain of NHERF1 in the micromolar range [194]. This example well illustrates the possibility to modulate the desired biological activity by changing substituents of the core skeleton. At the same time, it rises a not negligible problem of selectivity among the PDZ domains.

The growing interest for PDZ modulators over the past years is due to their vast therapeutic potential (Table 2). However, despite several hit-compounds have been discovered, none of them entered the clinic stages, suggesting that the optimization of initial hit-compounds is not trivial. Overall, the drug discovery community have the opportunity to step up to the challenge posed by the development of PDZ inhibitors as new treatments for still unsolved diseases.

**Table 2:**
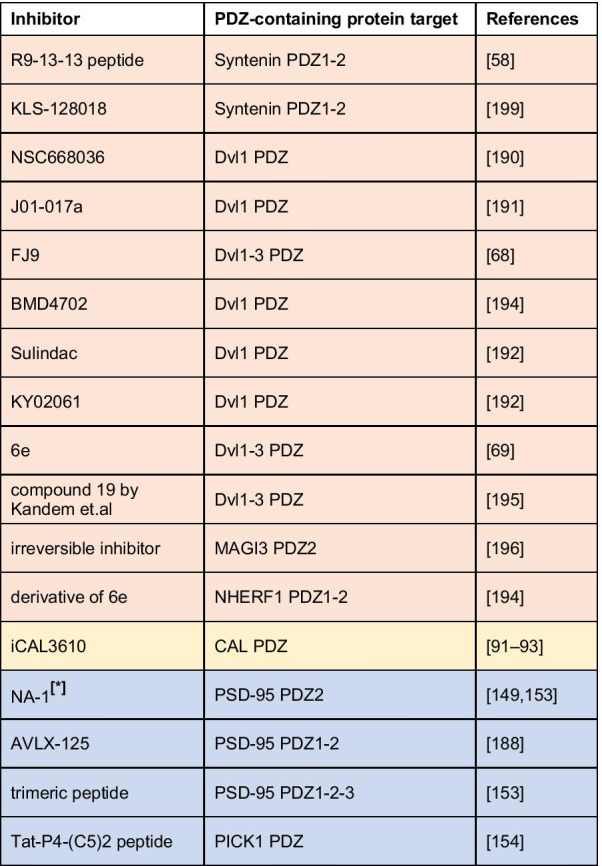
A summarizing list of inhibitors designed and developed in order to inhibit the binding of PDZ-containing proteins involved in different human pathologies (light red, yellow, and light violet respectively for cancer, cystic fibrosis, and nervous system disorders)

*Inhibitor in phase III trial

## Conclusions

PDZ domains are the most diffused structural and functional protein modules mediating protein–protein interactions, and they serve as fundamental elements in the construction of the cellular molecular machinery. Because of their importance in several molecular and physiological pathways, misregulation of PDZ-mediated interactions causes the onset of many pathologies. Thus, PDZ domains represent interesting drug targets and the inhibition of their interactions is an important pharmacological strategy to treat those pathologies.

A continuously increasing effort from the scientific community allowed the development of brilliant experimental strategies aimed to target PDZ domains. However, difficulties arose during the years to achieve this goal, ranging from the generally low binding selectivity of PDZ domains, which prevented the targeting of a specific PDZ domain, to the scarce efficiency of small molecules in inhibiting PDZ binding. As already described in paragraph 1.1 PDZ domains can be generally classified in three classes based on their binding specificity, depending on the sequence of the recognized protein. However, a proteomic analysis conducted on mouse PDZ domains demonstrated this classification to be superficial, specificity of PDZ domains being particularly broad [197], multiple residues, probably arranged in the binding pocket, driving selectivity. In particular, residues 0 and -2 were shown to possess key role in determining affinity, while residues -4, -3, -2 and -1 seem to orchestrate the selectivity of the recognition [197] with overlapping consensus sequences among the domain family. As an illustrative example of the complexity of successful inhibition of PDZ domains, in order to improve affinity and selectivity in targeting PDZ domain of CAL and avoid cross-reactivity, the design of a selective inhibitor of CFTR-CAL binding (showed in Fig. 3), required not just a “C-terminal core” (-4, -3, -2, -1, 0) to be taken into account, but also residues upstream (-9,-8,-7,-6,-5) were found to be fundamental [91].

Under this light, it appears clear that achieving a controlled selectivity in targeting of PDZ domain appears to be a difficult but crucial task, in order to decrease cross-reactivity and improve efficacy in therapies based on the inhibition of PDZ-containing proteins. Nevertheless, given the importance of PDZ domains in several human pathologies, further theoretical and experimental investigations are required in order to develop feasible and efficient drugs able to regulate and inhibit PDZ mediated interactions.

## Data Availability

Not applicable.
